# Relationships between students’ perceived campus walkability, mental health, and life satisfaction during COVID-19

**DOI:** 10.1038/s41598-024-65116-y

**Published:** 2024-06-21

**Authors:** Zhehao Zhang, Jinyun Wang, Haiming Wang, Jinxin Wu

**Affiliations:** 1 School of Architecture, Yantai University, Yantai, 264005 China; 2 People’s Hospital Affiliated to Shandong First Medical University, Jinan, 271199 China; 3 School of Architecture and Engineering, Yantai Institute of Technology, Yantai, 264003 China; 4 School of Architecture and Artistic Design, University of Science and Technology Liaoning, Anshan, 114051 China

**Keywords:** Perceived campus walkability, Socio-psychological environment, Academic performance, Mental health, Life satisfaction, Psychology and behaviour, Public health, Quality of life

## Abstract

Improving walkability in the campus environment and socio-psychological environments can promote students’ mental health and subjective well-being. This study aimed to propose a theoretical model to investigate the link of perceived campus walkability (PCW) with mental health and life satisfaction (LS), and to disentangle the mediating impact of socio-psychological environments and academic performance on this relationship, while simultaneously considering the effect of the COVID-19 pandemic. We applied structural equation modeling to analyze the data collected through a questionnaire survey conducted at six universities and colleges in Yantai, China. PCW had both direct and indirect positive effects on mental health and LS. However, indirect effects are greater than direct effects. Walking attitudes, social capital, and academic performance were critical to the relationship between PCW, mental health, and LS. Academic performance had the strongest indirect effect on mental health, while social capital had the strongest indirect effect on LS. We also found that during the COVID-19 pandemic, body mass index and family income were significantly correlated with mental health and LS. The findings indicate that campus planners and policymakers should improve PCW and support the socio-psychological environment to promote students’ mental and social health during situations like the COVID-19 pandemic.

## Introduction

Numerous studies have found that a high percentage of college students suffer from mental health disorders, such as stress, anxiety, and depression^1,2^. Studies have found that 12–43% of college and university students are diagnosed with anxiety^3,4^. This ratio has increased during the COVID-19 pandemic. One study found that nearly 30% of the Chinese students experienced mild and greater symptoms of anxiety during the COVID-19 pandemic^5^. Moreover, health worries caused by the fear of contracting the new coronavirus have led to a decrease in the general public's life satisfaction (LS), which is used to evaluate individuals’ quality of life^6^.

Building a walkable environment can effectively prevent mental health disorders and promote LS^7,8^. Many studies selected walkability features related to “D” variables (density, diversity, design, etc.) and explored their associations with health outcomes^9^. Researchers have optimized the widely adopted instrument of the Neighborhood Environment Walkability Scale (NEWS) and its abbreviated version (NEWS-A) to measure respondents’ perceived built environment walkability^10^. They found that streetscape greenery availability^11^, facility accessibility^12^, access to open spaces^13^, aesthetic quality^14^, and other perceived features related to walkability^15,16^ were significantly correlated with mental health and LS in the general population. Specifically, scholars have disclosed that campus walkability features, such as green and public spaces^17,18^, perceived naturalness^17^, environmental design qualities^19^, and other characteristics, can significantly promote students’ mental health and subjective well-being. One review suggested that campus environments benefit student health. It was concluded that built environments with aesthetics, transportation-related features, and natural environments with greenness, trees, etc. can significantly advance students’ health outcomes^20^.

Moreover, academia has substantiated that socio-psychological environments of walking attitudes, social capital, and place attachment significantly correlate with mental and social health. Liu et al. reported that walking attitudes are significantly correlated with mental and physical health states^21^. Hipp et al. found that perceived restorativeness strongly improved students’ quality of life^22^. Importantly, some studies have confirmed that socio-psychological factors can be critical in mediating the correlation between walkability, mental health, and subjective well-being. Li et al. and Mouratidis revealed that social cohesion and a sense of community are vital in mediating the association between walkability and mental and social well-being^23,24^. Notably, the relationship of the primary factor affecting students’ mental and social health—academic performance associated with learning experience, retention, and graduation rates—with campus walkability has been analyzed^25,26^.

Scholars have found that COVID-19 has significantly and negatively influenced students' academic lives, mental health, and LS in various countries^27,28^. Many Chinese scholars have investigated the relationship between the pandemic and students' health outcomes. Liu et al. reported that the pandemic has significantly and adversely affected students' physical and mental conditions^21^. Cao et al. indicated that the worry caused by the COVID-19 infection to relatives and friends was a vital risk factor for students' anxiety^5^. Rogowska et al. indicated that the pandemic had a significant detrimental effect on Polish students' stress, anxiety, and LS (Rogowska et al., 2020). Browning et al. studied students on seven U.S. campuses and investigated various psychosocial impacts of the pandemic on them. They found that all surveyed students were somehow affected by the pandemic and that sex, age, screen time, and other factors were strongly associated with students' psychological impact^29^. Moreover, one review article found that strict campus-closure strategies, travel bans, and a lack of interpersonal communication caused by the pandemic were the main causes of students' anxiety^30^. As numerous studies have highlighted the significant impact of the pandemic on college students’ health, it is imperative to consider the pandemic effect in this study.

Although academia has confirmed that walkable environments can promote mental health and LS, and that the pandemic can impede health outcomes, there are still three main research gaps. First, few studies have analyzed the link between PCW, mental health, and LS simultaneously and considered the pandemic. Second, the mediating effects of walking attitudes, social capital, and academic performance on this link during the pandemic are yet to be explored^29^. Although Liu et al. primarily investigated students’ relationship with perceived green spaces and their restoration and health^17^, they omitted the effect of mediators on these relationships. Moreover, although Liu et al. explored the mediating effect of walking behavior on the link of the perceived built environment with health^21^, they neglected the effect of social environment mediators widely verified in the literature on this association. Third, many strategies and guidelines for promoting students' well-being and mental health^19,31^ have been proposed based on a qualitative perspective without verification through evidence-based studies.

**Figure 1:**
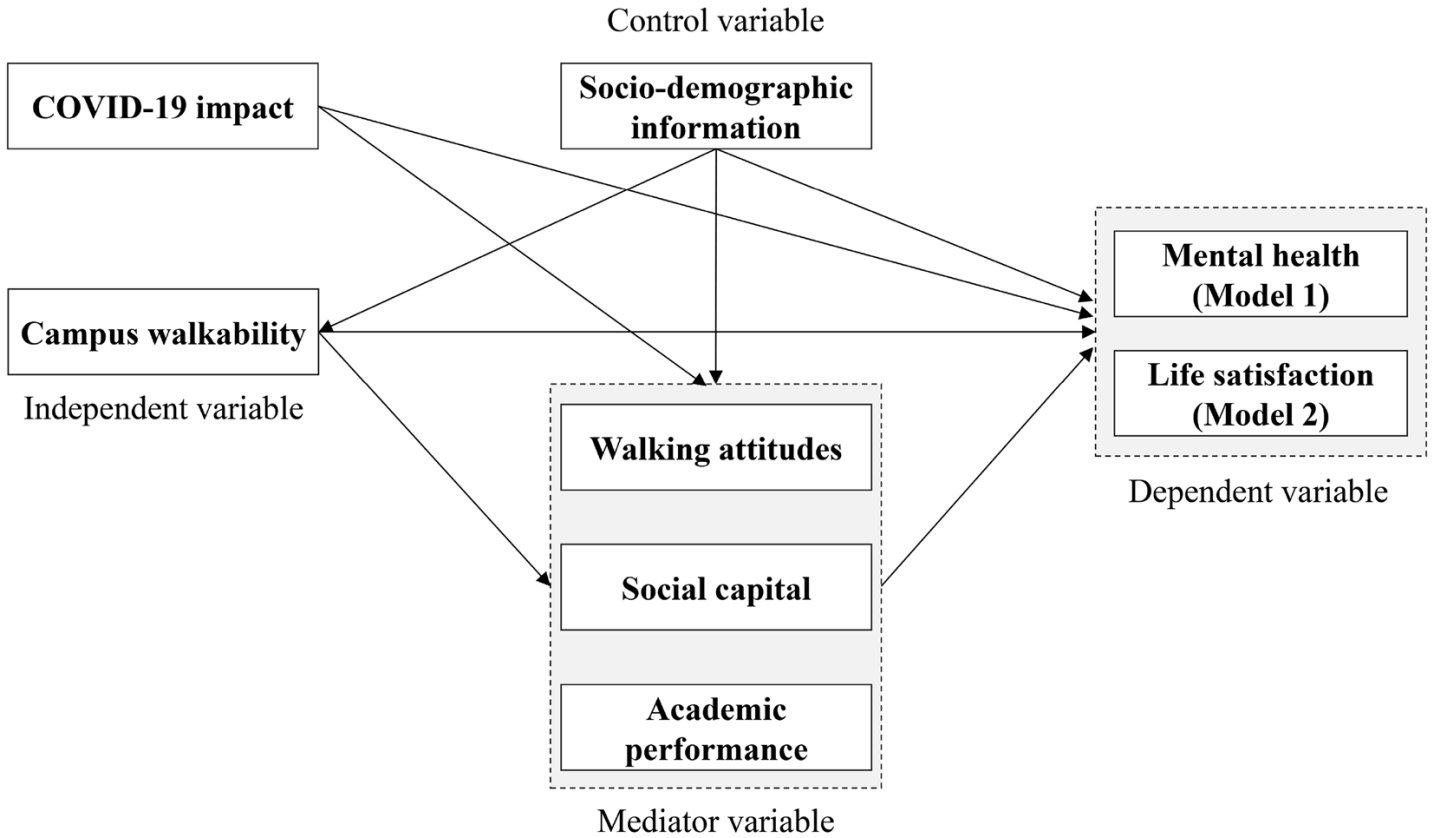
The conceptual model of this study.

This study fills these research gaps by presenting a conceptual model to explore relationships among PCW, mental health, and LS during the pandemic, to derive the environmental factors affecting students’ mental health, and LS to provide strategies to improve their health conditions. We used structural equation modeling (SEM) to analyze data collected through a questionnaire survey conducted at six Chinese campuses in Yantai (Fig. 1). We further investigated the role of the mediators of walking attitudes, social capital, and academic performance in the association between PCW, mental health, and LS. We intended to answer three critical questions: (1) What is the relationship between PCW, mental health, and LS? (2) To what degree does the mediating variable influence this correlation? and (3) What are the effects of the COVID-19 pandemic on mental health and LS?

We formulated the following hypotheses:

- (1) Based on previous urban studies^16,23^, we hypothesized that PCW would significantly impact mental health and LS in direct and indirect patterns.
- (2) Congruent with the finding of existing studies that social-psychological environments^21^ and academic performance^26^ are significantly correlated with perceived environments and health outcomes, we expected that walking attitudes, social capital, and academic performance would mediate the effect of PCW on mental health and LS.
- (3) As previously shown^21,27^, we hypothesized that the COVID-19 pandemic would negatively influence students’ mental health and LS.

This study makes three advances to the existing literature: (1) We comprehensively selected PCW, social-psychological environments, academic performance, and the COVID-19 pandemic impact as research variables to construct a research framework to disentangle the effect mechanism of PCW on mental health and LS simultaneously. (2) We revealed the relative importance of the mediating effects of walking attitudes, social capital, and academic performance on the relationship between PCW, mental health, and LS. (3) Based on these results, we present targeted strategies to design campus environments to promote students' mental and social health. The remainder of this paper is organized as follows. Section "Methods" describes the study area, variable collection, and data-analysis methods. Section "Results" presents the results of the SEM analysis. The findings of this study are presented in Section "Discussion". Finally, section "Conclusion" concludes the paper.

## Methods

### Study area

We conducted a cross-sectional survey with a comprehensive questionnaire at six universities and colleges in Yantai, China—Yantai University (YU), Ludong University (LU), Shandong Technology and Business University (STBU), Binzhou Medical University (BMU), Yantai Institute of Technology (YIT), and Yantai Vocational College (YVC)—to collect research data. Yantai has rich educational resources and diverse campuses. University campuses in Yantai can serve as specific areas to reflect on the current problems faced by Chinese university campuses. The chosen samples included university campuses, colleges, and higher education colleges in China and reflected Chinese college students’ daily needs. Moreover, the selected campuses have various functions and differ in size, construction period, form, and surrounding environments, which reflect the distinctive characteristics of Chinese campuses. The results and conclusions derived from this study can provide generalizability, suggestions, and references for campuses in other areas.

The survey was conducted in October 2022, during the COVID-19 pandemic. The pandemic became more severe during the questionnaire-collection period. The university has adopted strict lockdown and control measures. Students were not allowed to leave campuses. As instructors in Chinese universities were responsible for managing students’ daily lives and studies in their colleges, we contacted instructors at each of the six universities and colleges. They further helped us join WeChat groups at each college. Each group comprised students from different grades. We randomly selected students from different grades at the same rate and asked them to scan the QR code link to complete the questionnaire. Before completing the questionnaire, we informed respondents about the purpose of the study and asked them to complete it anonymously. The survey respondents were registered students living on campus. Each participant took an average of 10 min to complete the survey. The research ethics committees of each university and college reviewed and approved the study. The date and IRB-approval numbers were 05/10/2022 and YTUHR-20221005-001, respectively. Informed consent was obtained from all participants. Questionnaire responses were unmarked. We collected 3325 questionnaires. Owing to a lack of supervision and welfare, many students filled in questionnaires incorrectly or omitted some questions, and we excluded these invalid questionnaires to obtain a total of 1105 valid questionnaires, with a return rate of 33.23%. Basic information for each campus is presented in Table 1. We used these valid questionnaires to conduct the SEM analysis.

**Table 1:** The basic information of the six campuses.

University Campus	Land Area/10,000 m^2^	Number of conducted questionnaires	Number of valid questionnaires	Valid return rate (%)
YU	199.9	551	194	35.21
LU	230.0	672	215	31.99
STBU	90.9	525	178	33.90
BMU	106.7	563	172	30.53
YIT	42.0	498	163	32.73
YVC	100.1	516	183	35.47

### Research variables

The variables examined in this study consisted of five parts. (1) Dependent variables: perceived mental health and LS; (2) independent variable: PCW; (3) mediator variables: walking attitudes, social capital, and academic performance; (4) control variable: sociodemographic information; (5) perceived impact of the COVID-19 pandemic. All data were collected using questionnaires. We applied SEM to conduct a correlation analysis. All methods for measurement and analysis of the questionnaire conformed to relevant guidelines in existing studies^12,21,23,32,33^.

**Dependent variable**: The Patient Health Questionnaire (PHQ-9) was used to measure students' mental health. The PHQ-9, which is a straightforward and easy-to-use tool for measuring depressive symptoms, has been validated in various populations and settings^32,34^. It contains nine questions developed according to the principles of the Diagnostic and Statistical Manual of Mental Disorders, Version Four (DSMIV). The students applied a four-point Likert scale ranging from (0) strongly disagree to (3) strongly agree, to answer questions related to depression over the past two weeks. We then summed the scores for each question to obtain the total value. PHQ-9 scores can be divided into four levels: normal, mild, moderate, moderately severe, and severe^34^. The score was over 10, which indicated that students were diagnosed with major depression. We further derived one question from the guideline of OECD^35^ to measure students' perceived campus LS: "All things considered, I'm satisfied with my college life.” Students answered this question on a five-point Likert scale ranging from (1) strongly disagree to (5) strongly agree. This method is similar to Leyden et al.'s study, which also used one question to measure respondents' subjective well-being^12^.

**Independent variable**: This study measured the students' subjective perceptions of the physical environment. Based on the Abbreviated Neighborhood Environment Walkability Scale (NEWS-A)^10^, we estimated student PCW in relation to the physical environments of campuses. Since no offline researcher supervised and interpreted the questions, it was difficult for students to fill out the questionnaire online carefully if there were too many questions; therefore, we streamlined the questions for measurements. Several studies have reported that population density, destination accessibility, street connectivity, sidewalk conditions, environmental quality, aesthetics, and safety are significantly correlated with walkability and health^7,9^. However, owing to closed walls and mandatory accommodation policies, Chinese college students live and study on campus, whose population density cannot be easily changed^36,37^. Without access cards, non-college personnel are not allowed to enter the campus freely, and the internal security measures of the campus are relatively complete. Therefore, we excluded other elements associated with walkability such as population density and crime safety. Finally, we considered five questions related to the accessibility of public service facilities, street connectivity, sidewalk configuration, walking-environmental quality and aesthetics, and traffic safety. Moreover, existing studies have already validated the chosen questions as being closely related to health^23,33^. Students answered these questions on a five-point Likert scale ranging from (1) strongly disagree to (5) strongly agree. The PCW measured in this study was reliable with a Cronbach's alpha of 0.865.

**Mediator variable:** Scholars have confirmed that social capital can increase mental health and LS by enhancing communication with neighbors and strengthening a sense of community belonging^12^. Moreover, as Chinese college students mainly rely on walking to reach different destinations, good walking attitudes increase their willingness to walk on campus, increase their opportunities to meet and greet their classmates, and interact with the natural environment, thus improving their mental health and LS. Furthermore, social capital and walking attitudes have been found to significantly mediate the association between the perceived built environment, mental health, and LS, which has been previously validated^21,23^. Accordingly, we chose the socio-psychological environmental attributes of walking attitudes and social capital as the mediator variables. We first selected three items to assess walking attitudes using a five-level Likert scale ranging from (1) strongly disagree to (5) strongly agree. The reliability of the walking attitudes was acceptable. Social capital was measured using three questions. The students evaluated the items on a five-point Likert scale ranging from (1) strongly disagree to (5) strongly agree. Cronbach’s alpha for social capital was 0.840, indicating reliability. The main task of Chinese university students is to study, and their academic performance can affect their daily mental health, self-achievement, and campus satisfaction. Moreover, existing studies have confirmed that campus-built environments can significantly and positively promote academic performance^25,26^. Therefore, academic performance was selected as the third mediating variable. Students assessed their academic performance through the following item: “My current academic performance belongs to the category of,” using a five-point scale: (1) = 90 or above, (2) = 89–80, (3) = 79–70, (4) = 69–60, (5) = 59 or below.

**Socio-demographics and COVID-19 impact**: Socio-demographic information on age, grade, family income, and body mass index (BMI) was collected. Regarding the COVID-19 impact, we asked one question to measure the perceived COVID-19 impact on students’ lives: “The pandemic has adversely affected my college life.” Students answered this question on a five-point Likert scale ranging from (1) strongly disagree to (5) strongly agree.

### Data analysis

We first performed confirmatory factor analysis to verify the validity of the perceived independent and mediator variables using SPSS 25. We derived three factors, which were associated with five questions related to campus walkability questions, with three questions each about walking attitudes and social capital. The KMO is 0.863, *P < *0.001. The accumulated contribution rate was 66.20% (Table 2), indicating that the independent and mediating variables were verified in this study. Subsequently, we applied SEM to test the conceptual model and explore the relationship between the PCW, mental health, and LS using the AMOS software package. SEM can analyze the direct, indirect, and total effects of research variables on the outcome variable. We built two models related to the associated conceptual models to disentangle the mechanism of the correlation of the PCW with mental health and LS. Model 1 was used to investigate the relationship between PCW and mental health. Model 2 explored the association between the PCW and LS. Each model contained latent variables and different path analyses. All models compared the relative importance of the direct and indirect effects of PCW on the outcome variables. To obtain reliable data-analysis outcomes consistent with parameters set by existing studies using the SEM method^24,38^, we used bootstrapping with 1000 replications with the aim of estimating the significance of the levels of the analysis.

**Table 2:** The reliability test and validity test of the research variables.

	Reliability test		Validity test	
Independent variable	Cronbach’s alpha	Factor1	Factor2	Factor3
PCW	0.865			
The campus has a high facility accessibility (such as canteens, stores, coffee shops, etc.)		0.765		
The campus has a high street connectivity and multiple walking path options		0.841		
The campus has a high sidewalk configuration (sidewalk quality, sidewalk width, tidiness, etc.)		0.812		
The campus has a high walking environmental design quality (public spaces, street trees, street furniture, etc.)		0.824		
The campus has a high walking safety from traffic		0.593		
Mediator variable				
Waking attitudes	0.613			
I like walking on the campus			0.839	
I prefer to walk on the campus if accompanied by a companion			0.733	
I like walking because it is good for health			0.589	
Social capital	0.843			
I can always communicate and greet my classmates and friends on campus				0.866
It is easy to make friends on campus				0.799
I can always get help and suggestions from my classmates and friends on campus				0.793

The KMO of the validity test is 0.863, *P < *0.001.

## Results

Detailed descriptions of the collected data are as follows. Table 3 presents the socio-demographic characteristics of the participants. Table 4 shows the descriptive statistics for the research variables. Table 5 outlines the participants’ mental health status. We found that 42.26% of the students had mild and above mental health issues and 11.58% had major depressive disorder. The PHO-9 score measured in this study was reliable with a Cronbach's alpha of 0.868. The total PHQ-9 score was included as a mental health variable.

**Table 3:** Socio-demographic characteristics of the study sample.

Variables	Categories	N = 1105	%
Gender	Men	382	34.57
	Women	723	65.43
Grade	Freshman	393	35.57
	Sophomore	164	14.84
	Junior	250	22.62
	Senior	99	8.96
	Graduate	199	18.01
Income	Low	442	40.00
	Medium	626	56.65
	High	37	3.35
BMI	BMI (18.5–23.9)	723	65.43
	BMI (≤ 18.4)	215	19.46
	BMI (24–27.9)	118	10.68
	BMI (≥ 28)	47	4.25

**Table 4:** Descriptive statistics of the research variables.

Variable	Definition	Max	Min	Mean	Std
Dependent variable					
Mental Health (PHQ-9)	The sum score of nine questions related to mental health	27	0	4.49	4.45
Life satisfaction	All things considered, I'm very happy with my college life	5	1	3.40	0.86
Control Variable Socio-demographic Variables					
Sex	A dummy variable indicating whether the respondent is a man or woman (0 = women;1 = men)	1	0	0.35	0.48
Grade	Education level of the respondent (1 = freshman; 2 = sophomore; 3 = senior high school; 4 = associate degree; 5 = graduate	5	1	2.59	1.49
Income	Levels of annual family income (CNY) (1 low income = below 50,000; 2 middle income = 50,000–100,000; 3 high income = 100,000–250,000)	3	1	1.63	0.55
BMI	BMI = Weight (kg) / Height (m) ^ 2	41.67	14.84	21.12	3.34
Independent variable					
Perceived campus walkability					
Facility Accessibility	The campus has a high facility accessibility (such as canteens, stores, coffee shops, etc.)	5	1	3.59	0.84
Street connectivity	The campus has a high street connectivity and multiple walking path options	5	1	3.46	0.94
Sidewalk Configuration	The campus has a high sidewalk configuration (sidewalk quality, sidewalk width, tidiness, etc.)	5	1	3.48	0.93
Interesting Space and Environment Quality	The campus has a high walking environmental design quality (public spaces, street trees, street furniture, etc.)	5	1	3.34	0.88
Traffic Safety	The campus has a high walking safety from traffic	5	1	3.43	0.92
Impact of COVID-19 pandemic					
COVID-19 impact	The pandemic has adversely affected my college life	5	1	3.92	0.98
Mediator variable					
Waking attitudes	I like walking on the campus	5	1	3.02	1.13
	I prefer to walk on the campus if accompanied by a companion	5	1	2.55	1.14
	I like walking because it is good for health	5	1	3.72	1.13
Social capital	I can always communicate and greet my classmates and friends on campus	5	1	3.02	1.13
	It is easy to make friends on campus	5	1	2.55	1.14
	I can always get help and suggestions from my classmates and friends on campus	5	1	3.72	1.14
Academic performance	1 = 59 or below, 2 = 69–60, 3 = 79–70, 4 = 89–80, 5 = 90 or above	5	1	3.14	0.67

**Table 5:** Descriptive statistics of the respondents’ mental health condition.

Mental Health (PHQ-9)	N	%	Men (%)	Women (%)
Normal (0–4)	638	57.74	32.45	67.55
Mild (5–9)	339	30.68	37.76	62.24
Moderate (10–14)	88	7.96	35.23	64.77
Moderately severe (15–19)	31	2.81	41.94	58.06
Severe (20–27)	9	0.81	44.44	55.56
Overall	1105	100		

Model 1 tested the relationship between PCW and mental health, using walking attitudes, social capital, and academic performance as mediator variables. We found that the PCW had both direct and indirect relationships with mental health (X^2^/df = 3.070, CFI = 0.962, GFI = 0.969, RMSEA = 0.043, SRMR = 0.055), as shown in Tables 6 and 7. This implies that the model fit the data well and its validity was verified. Specifically, the proportion of the indirect effect to the total effect was 0.72, which was much higher than the direct effect of 0.28. The significance of the indirect effect of PCW on mental health was higher than that of the direct effect. The total effect is also significant, with the standardized coefficient and *P* value at   −  0.335 and *P < *0.05, respectively. Regarding the pathways of the indirect effect through the three mediator variables, we found that the significance of the mediating effect of academic performance on the relationship between PCW and mental health was the highest (*P < *0.01), and the estimate was − 0.716. The significance of the mediating effect of social capital on the relationship between PCW and mental health was at *P < *0.05. However, the significance of the mediating effect of walking attitude on the relationship between PCW and mental health was the lowest (*P < *0.1). Moreover, we found that PCW was significantly correlated with the three mediator variables. The significance and standardized coefficient of the relationship between PCW and social capital were the highest (*P < *0.01), and the standardized coefficient was 0.585. In contrast, the PCW's significance and standardized coefficient of correlation with academic performance were the lowest (*P < *0.01), and the standardized coefficient was 0.104. Walking attitudes, social capital, and academic performance were significantly negatively associated with mental health. Academic performance has the highest significant standardized coefficient of − 0.181, while walking attitudes have the lowest significant standardized coefficient of − 0.158.

**Table 6:** Standardized direct, indirect, and total effects of Model 1.

	PCW	Walking attitudes	Social capital	Academic performance	Mental health
Direct effects					
Sex	− 0.021	0.013	0.058*	**− **0.024	0.032
Grade	− 0.093**	− **0.053**^**a**^	0.089*	0.164*	**0.049**^**a**^
Income	0.064*	0.030	0.049	0.049	**− **0.028
BMI	− 0.094**	− 0.030	0.003	− 0.070*	0.038
PCW		0.318*	0.585**	0.104*	− 0.110*
COVID-19 impact		− 0.040	0.010	0.025	0.163**
Walking attitudes					− 0.105*
Social capital					− 0.219*
Academic performance					− 0.127**
Indirect effects					
Sex		**− **0.007	**− **0.012	-0.002	-0.005
Grade		− 0.030**	− 0.054**	-0.010**	-0.008
Income		0.020*	0.037*	0.007*	-0.038**
BMI		− 0.030**	− 0.055**	-0.010*	0.038**
PCW					-0.175**
COVID-19 impact					-0.001
Walking attitudes					
Social capital					
Academic performance					
Total effects					
Sex	**− **0.021	0.006	0.046	**− **0.027	**− **0.023
Grade	− 0.093**	− 0.083*	0.035	0.155*	**− **0.010
Income	0.064*	**0.050**^**a**^	0.087*	0.056	− 0.113*
BMI	− 0.094**	**− **0.060	**− **0.051	− 0.080*	0.029*
PCW		0.318*	0.585**	0.104*	− 0.335*
COVID-19 impact		**− **0.040	0.010	0.025	0.120**
Walking attitudes					− 0.158*
Social capital					− 0.290*
Academic performance					− 0.181**

Note: Significant are in value [bold].

^a^ < 0.1; * < 0.05; ** < 0.01; *** < 0.001.

**Table 7:** Indirect paths of Model 1.

Path analysis of mental health	Indirect effect		
Mental health level	Est	SE	P
PCW → Walking attitudes → mental health	**-0.623**^**a**^	0.335	0.086
PCW → Social capital → mental health	-0.777*	0.316	0.010
PCW → Academic performance → mental health	-0.716**	0.197	0.007
The proportion of the direct effect on the total effect	0.280*	0.090	0.012
The proportion of the indirect effect on the total effect	0.720**	0.090	0.008

Note: Significant are in value [bold].

^a^ < 0.1; * < 0.05; ** < 0.01; *** < 0.001.

The impact of COVID-19 has been correlated with poor mental health. The standardized coefficient of the total effect was 0.120, with *P < *0.01. Notably, the direct effect was insignificant, whereas the indirect effect was significant at *P < *0.01, with a standardized coefficient of 0.120. Regarding the relationship between socio-demographic factors and mental health, income and BMI were significantly correlated with mental health. The former shows a negative relationship, whereas the latter shows a positive relationship. Moreover, these two socio-demographic variables indirectly influenced mental health. Grade, income, and BMI were significantly related to PCW, and these three variables indirectly affected the mediator variables.

The PCW was positively associated with LS both directly and indirectly. We found that the model’s X^2^/df = 3.112, CFI = 0.964, GFI = 0.969, RMSEA = 0.044, SRMR = 0.045, as shown in Figure A2. This implies that Model 2 was also verified. As shown in Table 8, the significance of the total effect of PCW on LS is *P < *0.01, with a standardized coefficient of 0.570. The significance of the indirect effect of PCW on mental health was higher than that of the direct effect. Table 9 shows that the proportion of the indirect effect of PCW on LS is 0.779, which is much higher than that of the direct effect, with only 0.221. The path analysis of the indirect effect on LS revealed that social capital had the strongest indirect effect on the link between PCW and LS, with an estimate of 1.191 and *P* = 0.003. However, walking attitudes and academic performance influenced the relationship between PCW and LS (*P < *0.05) with estimates of 0.032 and 0.119, respectively. Consistent with the conceptual Model 1, we found that PCW was also positively and significantly correlated with the three mediator variables in Model 2. The significance and coefficient of the relationship between PCW and social capital were higher than those between walking attitudes and academic performance. Inconsistent with significant correlations between the three mediator variables and mental health, only social capital was positively and significantly correlated with LS.

**Table 8:** Standardized direct, indirect, and total effects of Model 2.

	PCW	Walking attitudes	Social capital	Academic performance	Life satisfaction
Direct effects					
Sex	− 0.021	0.013	0.057*	− 0.024	0.013
Grade	− 0.093**	− 0.053*	0.090*	0.164*	− 0.005
Income	0.064*	0.030	0.049	0.049	− 0.041
BMI	− 0.094**	− 0.030*	0.004	− 0.070*	0.011
PCW		0.318*	0.587*	0.104*	0.319*
COVID-19 impact		0.025	0.011	0.025	− 0.151**
Walking attitudes					0.033
Social capital					0.409**
Academic performance					0.001
Indirect effects					
Sex		− 0.007	− 0.012	− 0.002	0.012
Grade		− 0.030**	− 0.055**	− 0.010**	− 0.018
Income		0.020*	0.038*	0.007*	0.057**
BMI		− 0.030**	− 0.055**	− 0.010*	− 0.053*
PCW					0.250**
COVID-19 impact					0.003
Walking attitudes					
Social capital					
Academic performance					
Total effects					
Sex	− 0.021	0.006	0.045	− 0.027	0.025
Grade	− 0.093**	− 0.083*	0.035	0.155*	− 0.023
Income	0.064*	**0.050**^**a**^	0.086*	0.056	0.016
BMI	− 0.094**	− 0.060	− 0.051	− 0.080*	− 0.042
PCW		0.318*	0.587**	0.104*	0.570**
COVID-19 impact		− 0.040	0.011	0.025	− 0.148**
Walking attitudes					0.033
Social capital					0.409**
Academic performance					0.001

Note: Significant are in value [bold].

^a^ < 0.1; * < 0.05; ** < 0.01; *** < 0.001.

**Table 9:** Indirect paths of Model 2.

Path analysis	Indirect effect		
Life satisfaction	Est	SE	P
PCW → Walking attitudes → life satisfaction	0.332*	0.071	0.014
PCW → Social capital → life satisfaction	1.191**	0.065	0.003
PCW → Academic performance → life satisfaction	0.119*	0.053	0.028
The proportion of the direct effect on the total effect	0.221*	0.027	0.020
The proportion of the indirect effect on the total effect	0.779**	0.027	0.005

Note: Significant are in value [bold].

^a^ < 0.1; * < 0.05; ** < 0.01; *** < 0.001.

Different relationships exist between the COVID-19 pandemic, socio-demographics, and LS. We found that the pandemic significantly and directly inhibited LS. The significance of its effect on LS is at *P < *0.01, with the standardized coefficient = − 0.151. Notably, we did not find a significant total effect of socio-demographic variables on LS, whereas income and BMI significantly and indirectly influenced LS. Income was positively correlated with LS; its standardized coefficient and significance were 0.057 and *P < *0.01, respectively. In contrast, the BMI was negatively associated with LS. The significance of its indirect effect on LS is at *P < *0.05, with the standardized coefficient = -0.053. Congruent with the relationships between socio-demographics, PCW, and mediator variables in Model 1, the grade and BMI were negatively associated with PCW at *P < *0.01, with standardized coefficients of − 0.093 and − 0.094, respectively. Comparatively, income was positively correlated with PCW. The significance of its effect was at *P < *0.05, and the standardized coefficient was 0.064. Moreover, grade, BMI, and income indirectly influenced walking attitudes, social capital, and academic performance in model 2. Grade negatively influences them at *P < *0.01, with standardized coefficients being − 0.030, − 0.015, − 0.010, respectively. BMI also negatively affects the three mediators, with the standardized coefficient being − 0.030, − 0.055, and − 0.010, respectively. However, income positively influenced them at *P < *0.05, with standardized coefficients being 0.030, 0.055, and 0.010, respectively.

We further analyzed whether the effects of the PCW and mediating variables on mental health and LS were related to different campuses. Specifically, we constructed six SEM models for mental health and six models for LS. In each set of models, we set a variable of campus attributes, which reflected different campuses; that is, whether a respondent belonged to a certain campus which we denoted as 1 or whether the respondent did not belong to the campus, which we indicated with 0. Table 10 shows that there is no significant total effect of campus attributes on mental health and LS in any of the models (all the significances are at *P* > 0.05), which further indicates that there is no significant effect of campus attributes on the results drawn from this study, thus improving the generalizability of this study’s conclusions.

**Table 10:** Standardized direct, indirect, and total effects of Models 1 and 2 on six campuses.

	Whether belongs to YU	Whether belongs to LU	Whether belongs to STBU	Whether belongs to BWU	Whether belongs to YIT	Whether belongs to YVC
	Mental health (LS)	Mental health (LS)	Mental health (LS)	Mental health (LS)	Mental health (LS)	Mental health (LS)
Direct effects						
Sex	0.034 (0.013)	0.030 (0.014)	0.024 (0.020)	0.029 (0.012)	0.032 (0.013)	0.032 (0.013)
Grade	0.043 (− 0.007)	0.042 (-0.001)	0.051* (-0.007)	0.054^a^ (-0.004)	0.049^a^ (− 0.005)	0.049^a^ (-0.005)
Income	− 0.029 (− 0.041^a^)	-0.029 (-0.040^a^)	-0.027 (-0.042^a^)	-0.028 (-0.041^a^)	-0.028 (-0.041^a^)	-0.028 (-0.041^a^)
BMI	0.038^a^ (0.011)	0.036 (0.012)	0.038^a^ (0.010)	0.038 (0.011)	0.038 (0.011)	0.038 (0.011)
Campus attribute	0.045 (0.011)	-0.032 (0.017)	0.037 (-0.035)	-0.030 (-0.008)	-0.013 (-0.001)	-0.003 (0.013)
PCW	− 0.111** (0.319*)	-0.113** (0.321*)	-0.114** (0.323*)	-0.108** (0.320*)	-0.111** (0.319*)	-0.111** (0.320*)
COVID-19 impact	0.163* (− 0.151*)	0.163* (-0.151**)	0.160* (-0.149*)	0.162* (-0.151**)	0.163* (-0.151**)	0.163* (-0.150**)
Walking attitudes	− 0.104** (0.033)	-0.105* (0.033)	-0.110* (0.038)	-0.107** (0.033)	-0.105* (0.033)	-0.105** (0.033)
Social capital	− 0.219* (0.409**)	-0.217* (0.408**)	-0.212* (0.403**)	-0.218* (0.409**)	-0.219* (0.409**)	-0.218* (0.408**)
Academic performance	− 0.126** (0.001)	-0.127* (0.001)	-0.126* (0.001)	-0.126* (0.001)	-0.127* (0.001)	-0.127* (0.001)
Indirect effects						
Sex	− 0.005 (0.012)	-0.005 (0.011)	-0.004 (0.013)	-0.007 (0.017)	-0.005 (0.012)	-0.005 (0.012)
Grade	− 0.008 (− 0.020)	-0.007 (-0.020)	-0.007 (-0.020)	-0.003 (-0.027)	-0.008 (-0.018)	-0.008 (-0.018)
Income	− 0.038** (0.057*)	-0.038** (0.057*)	-0.038** (0.057*)	-0.038** (0.057*)	-0.039** (0.058*)	-0.038** (0.057*)
BMI	0.038* (− 0.053*)	0.038* (-0.053*)	0.038* (-0.053*)	0.038* (-0.053*)	0.036* (-0.049*)	0.038* (-0.053*)
Campus attribute	-0.003 (0.013)	0.004 (-0.009)	0.002 (-0.011)	-0.027^a^ (0.053*)	0.032^a^ (-0.046*)	-0.010 (0.002)
PCW	-0.175* (0.250**)	-0.174* (0.250**)	-0.173* (0.250**)	-0.174* (0.250**)	-0.174* (0.250**)	-0.175* (0.250**)
COVID-19 impact	-0.001 (0.003)	-0.001 (0.003)	-0.002 (0.005)	-0.001 (0.003)	-0.001 (0.003)	-0.001 (0.003)
Walking attitudes						
Social capital						
Academic performance						
Total effects						
Sex	0.029 (0.026)	0.025 (0.025)	0.020 (0.034)	0.022 (0.029)	0.027 (0.025)	0.027 (0.025)
Grade	0.035 (-0.026)	0.035 (-0.021)	0.044 (-0.027)	0.051 (-0.031)	0.041 (-0.023)	0.041 (-0.023)
Income	-0.067* (0.016)	-0.067* (0.017)	-0.066* (0.015)	-0.066* (0.016)	-0.067* (0.017)	-0.066* (0.016)
BMI	0.075* (-0.042)	0.074* (-0.041)	0.076* (-0.043)	0.075* (-0.042)	0.074* (-0.038)	0.076* (-0.042)
Campus attribute	0.041 (0.025)	-0.028 (0.008)	0.039 (-0.046)	-0.057^a^ (0.045)	0.019 (-0.047^a^)	-0.013 (0.016)
PCW	-0.286** (0.569*)	-0.286** (0.571*)	-0.287** (0.573*)	-0.282** (0.570*)	-0.285** (0.569*)	-0.286** (0.571*)
COVID-19 impact	0.162* (-0.147**)	0.162* (-0.148**)	0.159* (-0.144**)	0.161* (-0.148**)	0.162* (-0.148**)	0.161* (-0.147**)
Walking attitudes	-0.104** (0.033)	-0.105* (0.033)	-0.110* (0.038)	-0.107** (0.033)	-0.105* (0.033)	-0.105** (0.033)
Social capital	-0.219* (0.409**)	-0.217* (0.408**)	-0.212* (0.403**)	-0.218* (0.409**)	-0.219* (0.409**)	-0.218* (0.408**)
Academic performance	-0.126** (0.001)	-0.127* (0.001)	-0.126* (0.001)	-0.126* (0.001)	-0.127* (0.001)	-0.127* (0.001)

Note: Significant are in value [bold].

^a^ < 0.1; * < 0.05; ** < 0.01; *** < 0.001. Values in parentheses represent the analyzing result of LS. We only aggregated the results related to mental health and LS for simplicity across the six campuses. We also checked the rationality of the fitness of the models’ results, which we omit for implicity.

## Discussion

### Relationships between the PCW and mental health and LS

To achieve the research goal of proposing and applying a conceptual model to investigate the association among the factors of PCW, mental health, and LS during COVID-19 and to explore the mediating effect of socio-psychological environments and academic performance on this correlation, we used a questionnaire survey to measure students’ PCW, walking attitudes, social capital, and mental health and LS. We applied the SEM method to disclose the pathways between the PCW, mental health, and LS. Path analysis compared the direct, indirect, and total effects of PCW on mental health and LS and the relative importance of the mediators’ mediating effects. The results derived from this study indicate various links among the PCW, three mediator variables, mental health, and LS. These empirical findings verify the conceptual model and research hypotheses.

We found that PCW significantly influenced mental health and LS, both directly and indirectly. Notably, indirect effects impacted mental health and LS more than the direct effects did. This means that a walkable campus environment with diverse land uses, high street connectivity, good environmental quality, and pedestrian facilities can encourage students to walk and engage in social activities on campus to maintain good mental health. Simultaneously, different campus activities can be used to foster friendships between college students and their peers, satisfaction in personal relationships, increased campus attachment, and social capital, which indirectly improve students’ mental health and LS. Therefore, enhancing campus walkability through specific objective physical and environmental interventions is imperative. This reinforces the findings of previous studies. Ding et al. reviewed 19 articles and confirmed that campus-built environment walkability of aesthetics, street connectivity, and sidewalk configurations were significantly correlated with students’ health conditions^20^. Evidence-based studies have been conducted in different regions in which the SEM analysis method has been applied. These studies have also corroborated that perceived built environment walkability can positively influence mental health and LS, both directly and indirectly^39,40^. For instance, Liu et al. found that students' PCW, especially accessibility, significantly promoted mental and physical states^21^, whereas Li et al. and Jun's team confirmed that perceived walkability could promote mental and social health^23,33^.

Different mediator variables presented various significant and positive mediating effects on the relationship between PCW, mental health, and LS. This means that improving walking attitudes, social capital, and academic performance promote the positive effects of PCW on mental health and LS. Importantly, we observed that the proportion of the indirect effect of the three mediators on the total effect was much higher than that of the direct effect. The actual influence mechanism is students' PCW, which first affects their perceived social and psychological environments and, second, their mental and social well-being. Specifically, we observed that the indirect effect of academic performance on the relationship between PCW and mental health was the highest. This indicates that Chinese university students often regard improving their academic performance as their primary goal. Faced with fierce competition in postgraduate exam preparation and employment pressure, college students face a more significant burden of study accompanied by mental stress; therefore, academic performance more significantly impacts students’ mental health. This finding is also strengthened by Cao et al.^5^ who found that worrying about academic delays was significantly correlated with mental health. Furthermore, we found that PCW significantly promotes academic performance. This finding is consistent with those of previous studies^25,26^. Hajrasouliha also reported that students' perceptions of campus environmental features were significantly correlated with their retention, graduation rates, and academic performance^25^. Social capital contributed the most to the association between PCW and LS. This finding is congruent with expectations because individuals greet each other, make friends, and get help from friends and classmates as components of social capital, which can help students enhance their campus attachment and social connectedness and improve their LS. This finding corroborates the argument echoed in other studies that social capital plays a vital role in the relationship between perceived environmental walkability and LS^23,41^. Consistent with the findings of the existing urban study^21^, we found that walking attitudes mediated the relationship between PCW, mental health, and LS to different degrees.

This study also found that the COVID-19 pandemic was significantly and negatively correlated with mental health and LS. Specifically, this effect directly influences the correlation. This finding was consistent with our expectations. The pandemic has directly affected students' college lives in two ways. First, college students fear the pandemic and worry about being infected by patients close to them. Therefore, the pandemic has directly affected students' mental well-being. Second, owing to the strict prevention and lockdown policies of the pandemic, students were prohibited from leaving campuses, which negatively affected their daily lives, increasing their mental stress and decreasing their LS. This finding is congruent with those of previous studies. Browning et al. found that the pandemic essentially affected all students, and more than half of them suffered from high levels of psychological impact^29^. Several empirical studies have found that the COVID-19 pandemic has significantly and negatively influenced students' health and well-being. Liu et al. found that the pandemic affected the physical and mental states of Chinese college students^21^. Rogowska et al. found that the impact of COVID-19 was strongly correlated with depression and anxiety in Polish students^2^. Regarding the total effect of students' socio-demographics on mental health and LS, we found that students with higher family incomes reported lower mental health. This means that students from high-income families enjoy better material conditions and are more likely to meet their daily needs, thus reducing mental stress. Moreover, students with higher BMI tended to have weaker mental states. This is because college students who are overweight or obese often have sedentary habits and are reluctant to go outside their dormitories for physical or social activities after school hours, forming a more monotonous and lonelier lifestyle that hurts their mental health.

### Practical interventions

Based on the findings derived from this study, we present targeted suggestions and interventions to promote students' mental and social health. First, campus planners should improve students' PCW through interventions for an objective built environment, such as improving accessibility to stores, canteens, and other public service facilities and enhancing land-use diversity, street connectivity, and sidewalk configurations. Existing studies have corroborated the finding that objectively measured built environment walkability can significantly correlate with walkability of the perceived built environment^33,42^. Second, university managers and policymakers should conduct thematic activities in different colleges to inform students about the distribution of facilities and infrastructure on campus, and about areas with high environmental quality and walkability to improve their perceptions and awareness of campus environmental walkability. Third, regarding the positive mediator effects of walking attitudes, social capital, and academic performance on the association between PCW, mental health, and LS, university governors should implement targeted policies and intervention programs to promote students' awareness of the importance of the social and psychological environments of a campus in terms of active walking attitudes, deep social connectedness, and good academic performance. These interventions can be disseminated through offline educational seminars or online platforms, such as WeChat. Finally, appropriate strategies should be implemented to reduce students' fear of COVID-19. This suggestion is critical because when the COVID-19 pandemic fades and other epidemics are confronted, college students will have similar psychologically adverse effects; therefore, reducing students' psychological impact through targeted strategies will promote their mental health and LS.

### Limitations

This study had some limitations. First, the research model developed in this study was verified using data from six campuses in Yantai, China. Owing to differences between the environments of campuses in tropical and frigid regions and those of the campuses selected by this study, whether the findings are suitable for application in campuses in other regions merits further investigation. Second, owing to limited resources and students not liking to fill out a questionnaire with many questions without supervision, especially several questions related to one theme, this study only considered five critical aspects closely associated with campus-environment walkability. However, other factors affecting walkability, such as street amenities and green visibility, were not considered. Therefore, to enhance the validity of the conceptual model, further studies should be conducted to evaluate the omitted features of this study and comprehensively examine various pathways between PCW, social-psychological environments, and mental and social health.

## Conclusion

This study systematically disentangled the direct, indirect, and total effects of PCW on students' mental health and LS. It also investigated the effects of the COVID-19 pandemic and mediator variables of walking attitudes, social capital, and academic performance on the association between PCW, mental health, and LS. We found that PCW had significant direct and indirect effects on mental and social health. However, the indirect effect was stronger than the direct effect. The three mediator variables were critical to relationships among PCW, mental health, and LS. Specifically, academic performance had a significant mediating effect on mental health. In contrast, social capital plays the most crucial mediating role in LS. Moreover, the COVID-19 pandemic significantly and negatively influenced mental health and LS, whereas BMI and family income significantly correlated with mental health and LS. These conclusions reinforce the findings of previous studies and provide concrete evidence for campus planners and policymakers to adopt appropriate measures for improving students' mental and social health.

### Supplementary Information

Supplementary Information.

## Data Availability

The datasets generated and analyzed during the current study are not publicly available due to they contain non-public data. But they are available from the corresponding author upon reasonable request.
