# Supplementary material for: Relationships between students’ perceived campus walkability, mental health, and life satisfaction during COVID-19
Source: Sci Rep. 2024 Jun 21;14:14333. doi: 10.1038/s41598-024-65116-y (PMC11192732; doi:10.1038/s41598-024-65116-y)
Supplement: Supplementary file 1 — Supplementary Information. [file 41598_2024_65116_MOESM1_ESM.docx]

**
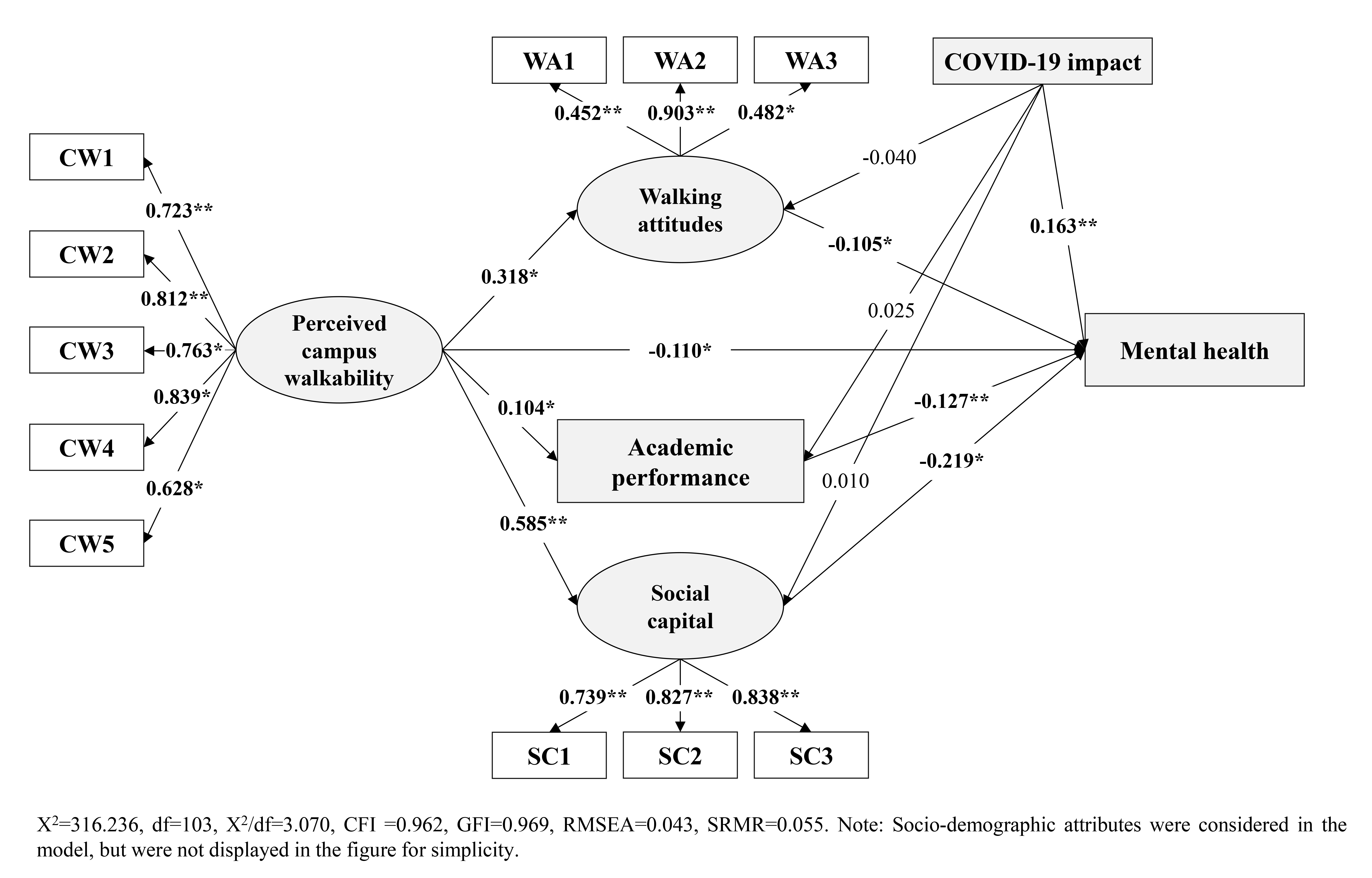
Appendix**


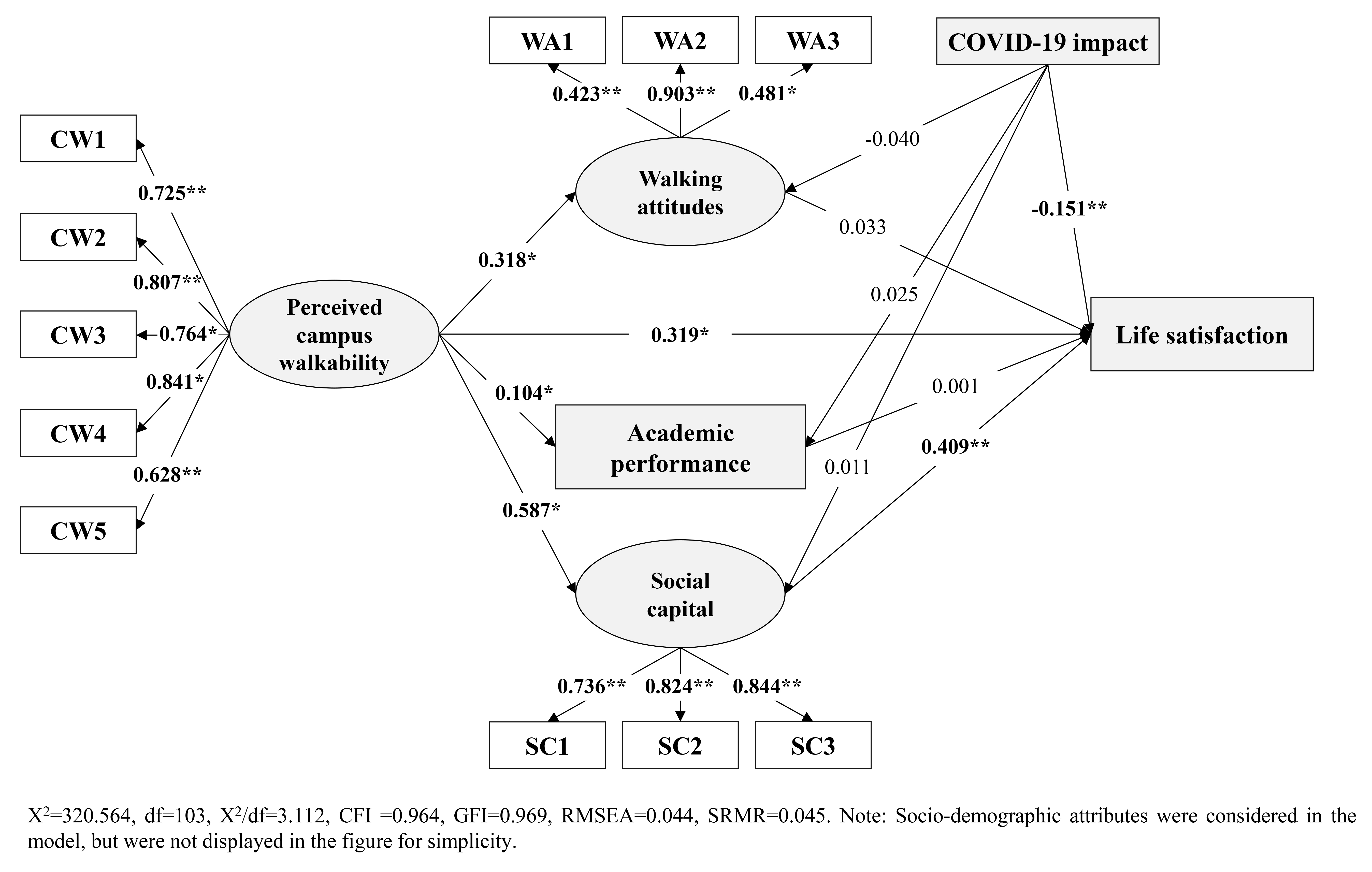
**Figure A1** Results of Model 1.

**Figure A2** Results of Model 2.

**Survey questionnaire**

1. Gender: **[1]** Men **[2]** Women

2. Grade: **[1]** Freshman **[2]** Sophomore **[3]** Junior **[4]** Senior **[5]** Graduate

3. Family Income (CNY): **[1]** Low (below 50,000) **[2]** Medium (50,000-100,000) **[3]** Junior (100,000-250,000)

4. Your campus name: **[1]** Yantai University **[2]** Ludong University **[3]** Shandong Technology and Business University **[4]** Binzhou Medical University **[5]** Yantai Institute of Technology **[6]** Yantai Vocational College

5. Height (m): ______

6. Weight (Kg): ______

***Campus environment walkability***

7. The campus has a high facility accessibility (such as canteens, stores, coffee shops, etc.).

**[1]** Strongly disagree **[2]** Disagree **[3]** Neutral **[4]** Agree **[5]** Strongly agree

8. The campus has a high street connectivity and multiple walking path options.

**[1]** Strongly disagree **[2]** Disagree **[3]** Neutral **[4]** Agree **[5]** Strongly agree

9. The campus has a high sidewalk configuration (sidewalk quality, sidewalk width, tidiness, etc.)

**[1]** Strongly disagree **[2]** Disagree **[3]** Neutral **[4]** Agree **[5]** Strongly agree

10. The campus has a high walking environmental design quality (public spaces, street trees, street furniture, etc.)

**[1]** Strongly disagree **[2]** Disagree **[3]** Neutral **[4]** Agree **[5]** Strongly agree

11. The campus has a high walking safety from traffic.

**[1]** Strongly disagree **[2]** Disagree **[3]** Neutral **[4]** Agree **[5]** Strongly agree

***Impact of COVID-19 pandemic***

12. The pandemic has adversely affected my college life.

**[1]** Strongly disagree **[2]** Disagree **[3]** Neutral **[4]** Agree **[5]** Strongly agree

***Walking attitudes***

13. I like walking on the campus.

**[1]** Strongly disagree **[2]** Disagree **[3]** Neutral **[4]** Agree **[5]** Strongly agree

14. I prefer to walk on the campus if accompanied by a companion.

**[1]** Strongly disagree **[2]** Disagree **[3]** Neutral **[4]** Agree **[5]** Strongly agree

15. I like walking because it is good for my health.

**[1]** Strongly disagree **[2]** Disagree **[3]** Neutral **[4]** Agree **[5]** Strongly agree

***Social capital***

16. I can always communicate and greet my classmates and friends on campus.

**[1]** Strongly disagree **[2]** Disagree **[3]** Neutral **[4]** Agree **[5]** Strongly agree

17. It is easy to make friends on campus.

**[1]** Strongly disagree **[2]** Disagree **[3]** Neutral **[4]** Agree **[5]** Strongly agree

18. I can always get help and suggestions from my classmates and friends on campus.

**[1]** Strongly disagree **[2]** Disagree **[3]** Neutral **[4]** Agree **[5]** Strongly agree

***Academic performance***

19. My current academic performance belongs to the category of___.

**[1]** 59 or below **[2]** 69-60 **[3]** 79-70 **[4]** 89-80 **[5]** 90 or above

***Mental health***

Over the last two weeks, how often have you been bothered by any of the following problems on campus?

20. Little interest or pleasure in doing things.

**[1]** Not at all **[2]** Several days **[3]** More than half the days **[4]** Nearly every day

21. Feeling down, depressed, or hopeless.

**[1]** Not at all **[2]** Several days **[3]** More than half the days **[4]** Nearly every day

22. Trouble falling or staying asleep or sleeping too much.

**[1]** Not at all **[2]** Several days **[3]** More than half the days **[4]** Nearly every day

23. Feeling tired or having little energy.

**[1]** Not at all **[2]** Several days **[3]** More than half the days **[4]** Nearly every day

24. Poor appetite or overeating.

**[1]** Not at all **[2]** Several days **[3]** More than half the days **[4]** Nearly every day

25. Little interest or pleasure in doing things.

**[1]** Not at all **[2]** Several days **[3]** More than half the days **[4]** Nearly every day

26. Feeling bad about yourself- or that you are a failure or have let yourself or your family down.

**[1]** Not at all **[2]** Several days **[3]** More than half the days **[4]** Nearly every day

27. Trouble concentrating on things, such as reading the book or watching videos on computers.

**[1]** Not at all **[2]** Several days **[3]** More than half the days **[4]** Nearly every day

28. Moving or speaking so slowly that other people could have noticed? Or the opposite, being so fidgety or restless that you have been moving around a lot more than usual.

**[1]** Not at all **[2]** Several days **[3]** More than half the days **[4]** Nearly every day

29. Thoughts that you would be better off dead or hurting yourself in some way.

**[1]** Not at all **[2]** Several days **[3]** More than half the days **[4]** Nearly every day

***Life satisfaction***

30. All things considered, I'm very happy with my college life.

**[1]** Strongly disagree **[2]** Disagree **[3]** Neutral **[4]** Agree **[5]** Strongly agree
